# Effects of Plant Diversity, Functional Group Composition, and Fertilization on Soil Microbial Properties in Experimental Grassland

**DOI:** 10.1371/journal.pone.0125678

**Published:** 2015-05-04

**Authors:** Tanja Strecker, Romain L. Barnard, Pascal A. Niklaus, Michael Scherer-Lorenzen, Alexandra Weigelt, Stefan Scheu, Nico Eisenhauer

**Affiliations:** 1 J.F. Blumenbach Institute of Zoology and Anthropology, Georg August University Göttingen, Göttingen, Germany; 2 INRA, UMR1347 Agroécologie, Dijon, France; 3 ETH Zurich, Zurich, Switzerland; 4 Institute of Evolutionary Biology and Environmental Studies, University of Zurich, Zurich, Switzerland; 5 Chair of Geobotany, Faculty of Biology, University of Freiburg, Freiburg, Germany; 6 Institute for Biology, University Leipzig, Leipzig, Germany; 7 German Centre for Integrative Biodiversity Research (iDiv) Halle-Jena-Leipzig, Leipzig, Germany; University of Oxford, UNITED KINGDOM

## Abstract

**Background:**

Loss of biodiversity and increased nutrient inputs are two of the most crucial anthropogenic factors driving ecosystem change. Although both received considerable attention in previous studies, information on their interactive effects on ecosystem functioning is scarce. In particular, little is known on how soil biota and their functions are affected by combined changes in plant diversity and fertilization.

**Methodology/Principal Findings:**

We investigated the effects of plant diversity, functional community composition, and fertilization on the biomass and respiration of soil microbial communities in a long-term biodiversity experiment in semi-natural grassland (Jena Experiment). Plant species richness enhanced microbial basal respiration and microbial biomass, but did not significantly affect microbial specific respiration. In contrast, the presence of legumes and fertilization significantly decreased microbial specific respiration, without altering microbial biomass. The effect of legumes was superimposed by fertilization as indicated by a significant interaction between the presence of legumes and fertilization. Further, changes in microbial stoichiometry (C-to-N ratio) and specific respiration suggest the presence of legumes to reduce N limitation of soil microorganisms and to modify microbial C use efficiency.

**Conclusions/Significance:**

Our study highlights the role of plant species and functional group diversity as well as interactions between plant community composition and fertilizer application for soil microbial functions. Our results suggest soil microbial stoichiometry to be a powerful indicator of microbial functioning under N limited conditions. Although our results support the notion that plant diversity and fertilizer application independently affect microbial functioning, legume effects on microbial N limitation were superimposed by fertilization, indicating significant interactions between the functional composition of plant communities and nutrient inputs for soil processes.

## Introduction

Loss of biodiversity and increased nutrient inputs are two of the most crucial anthropogenic impacts on Earth’s biosphere [1,2]. Many studies have investigated the effects of species loss and eutrophication on ecosystem functioning; however, usually these factors have been considered in isolation. Knowledge of how these factors interactively influence ecosystem functions, such as decomposition and element cycling, is incomplete (but see e.g. [3,4]).

Plant diversity plays an important role for ecosystem functions, such as primary productivity [5,6] and its temporal and spatial stability [7–9]. Diverse grassland plant communities are more productive than plant communities with low diversity [6,10]. Positive effects of species diversity on plant productivity in turn increase the input of organic carbon (C) to the soil, e.g., by producing more root exudates and shoot and root litter, thereby enhancing resource supply to decomposers [11,12]. In particular, grasses may lead to high rates of soil microbial respiration and biomass due to their dense root system and high root exudation rates compared to other plant functional groups [13,14].

Microbial communities are known to respond to the identity and diversity of C substrates secreted by plant roots [11,15,16]. As different plant species provide different biochemical compounds [17], higher plant diversity is likely to improve the nutrition of microorganisms. Thus, microorganisms may not only profit from higher quantity, but also from higher variety of plant-derived resources in species-rich plant communities [11] and from lower temporal variability of C supply [18]. Consequently, microbially-driven processes, such as decomposition and element cycling, are affected by plant diversity [19]. Previous studies found microbial biomass to increase with increasing plant diversity [12,14,20], thereby also modifying biogeochemical cycles [4,12]. However, previous studies also stressed the importance of certain plant functional groups for the composition and functioning of soil organisms [21,22]. For instance, N fixation by rhizobia associated with legumes and high root biomass of grasses have been identified as major drivers of soil communities [22,23].

Given the significant role of N in shaping the composition of terrestrial ecosystems [24], anthropogenic N inputs may alter the relationship between plant diversity and ecosystem functioning [25] as well as interactions between plants and soil organisms [4,26]. Soil microorganisms are generally C-limited [27–29] and drive soil N transformations that, for a large part, require easily accessible C [28]. On the other hand, plants are typically N- and/or P limited [30]. N addition therefore generally increases primary productivity and organic matter input to the soil [31,32], and can directly contribute to microbial growth and activity [33], thereby leading to enhanced competition between plants and microorganisms for N [34]. However, N addition can also adversely affect soil microbial biomass and activity [33]. A number of mechanisms have been proposed to explain effects of increased N input on soil microbial growth, including soil acidification [35,36] as well as leaching of nutrients such as magnesium and calcium [24,33]. This leads to uncertainty on the overall outcome for soil microbial properties, especially when considering N addition in interaction with other environmental changes.

We investigated effects of plant diversity and community composition, fertilizer addition, and their interactions on the activity (respiration) and biomass of soil microorganisms in temperate grassland. At the Jena Experiment field site, where we conducted this study, the positive effect of fertilizer on primary productivity was slightly strengthened by plant species richness [37]. However, effects of fertilization mainly occurred in plots without legumes [37],suggesting significant interactive effects of plant community composition and fertilization on soil microorganisms. Specifically, we tested the following hypotheses:

1. Plant species and functional group richness increase soil microbial respiration and biomass due to increased resource supply by plants.
2. Plant functional groups (grasses, small herbs, tall herbs, legumes) differently affect soil microorganisms due to group-specific functional traits.
3. Fertilization increases soil microbial biomass and respiration by enhancing plant productivity.
4. Effects of plant diversity and community composition on soil microbial respiration and biomass are strengthened by fertilization.

## Materials and Methods

### Ethic statement

Soil and plant biomass sampling was conducted with the permission of the city council of Jena, Germany.

### Study site

The experiment was performed on the field site of the Jena Experiment, a long-term biodiversity study focusing on the role of biodiversity for ecosystem functioning in semi-natural temperate grassland [38]. The study site is situated in the floodplain of the Saale River near the city of Jena (Thuringia, Germany, 50°55`N, 11°35`E, 130 m a. s. l.). Mean annual temperature is 9.3°C and mean annual precipitation is 587 mm. Prior to establishment of the Jena Experiment in May 2002 the site had been used as arable field for about 40 years. The plant communities established in the Jena Experiment were assembled from plant species typical for hay meadows in Central Europe.

### Experimental design

We established model grassland communities from a pool of 60 plant species differing in two aspects of plant diversity. The experiment included a gradient of plant species richness of 1, 2, 4, 8, 16 and 60 plant species and a gradient of plant functional group richness of 1, 2, 3 and 4 different plant functional groups (for details see [38]). Plant species were ascribed to functional groups using cluster analysis based on above- and belowground morphological traits, phenological traits, and N_2_ fixation [38]. The 60 species were grouped into grasses (16 species), small herbs (12 species), tall herbs (20 species), and legumes (12 species).

The experiment consists of 82 plots of 20 × 20 m. Plots are mown twice a year in June and September and weeded in April and July to maintain the target plant species composition. Plots were grouped into four blocks with two blocks sampled in the present study. Each block contains an equal number of plots of plant species and plant functional group richness levels. For more detailed information on the experimental design see [38].

### Fertilizer treatment and aboveground plant biomass sampling

Within each plot of 20×20 m, two subplots of 1.6 × 4.0 m each were established; fertilizer was added to one of the subplots as mineral NPK pellets (100 kg N ha^-1^, 44 kg P ha^-1^, 83 kg K ha^-1^) in early spring (April 2006 and March 2007) and after the first mowing (June 2006 and June 2007); control subplots were kept unfertilized. Plots were mown twice a year during the growing season (June and September) at approximately 3 cm above soil surface. The cut material was removed from the plots. Mowing, fertilizing, and weeding were carried out block-wise, and the block effect was included in the statistical model. For more details see [37].

It is known that fertilization can reduce plant species richness, especially in the long-term [24,39]. To test whether fertilization altered plant species richness in the present study, plant species number was recorded in 2008 on plots with sown diversity levels of 1, 2, 4, 8, and 16 plant species. Species richness was derived from species-specific frequency measurements in 30 quadrats of 10 x 10 cm in size within the core area of 1x1 m in fertilized and unfertilized subplots (S1 Fig). Indeed, fertilization slightly reduced plant species richness compared to unfertilized subplots (F = 10.54, P<0.05), but on average only by 0.52 ± 0.59 species. Thus, we consider changes in plant diversity due to fertilization to be negligible. Additionally, we found a significant positive effect of realized species richness on microbial biomass C in both fertilized and unfertilized subplots (F = 11.43, P<0.05 and F = 4.5, P<0.05, respectively), confirming the results of the model using design variables. We are aware that high plant diversity cannot be sustained in fertilized grasslands in the long-term, but in this experiment synchronous maintenance of both fertilization and high plant diversity was possible because plant species loss due to fertilization was slow enough [37].

Notably, the design variables of our model, in particular presence of plant functional groups, were not significantly influenced by fertilization. This was confirmed by additional statistical analyses (GLM), where we tested the effect of fertilization on the presence of plant functional groups. Even the presence of legumes, which was most reduced in the framework of this experiment compared to the other plant functional groups, was not significantly influenced by fertilization (for the effect of fertilization on the presence of legumes: F = 2.59, p>0.1; average reduction of legume species was only 0.19 ± 0.27 species).

### Soil and aboveground plant biomass sampling

Soil samples were taken in June 2008 in control and fertilized subplots in each of the plots of blocks 1 and 2. Eight samples were taken per subplot with a soil corer (1.5 cm diameter, 15 cm deep), pooled and transferred to the laboratory. Roots and soil animals were picked by hand and the samples sieved through 2 mm mesh. Aboveground plant biomass was harvested subplot-wise in one randomly placed 0.2 × 0.5 m area, dried (70°C, 48h) and weighed [37].

### Soil microbial biomass, C-to-N ratio, and respiration

Microbial biomass C (MBC) was measured by substrate-induced respiration (SIR; see below) and chloroform fumigation extraction (CFE); soil microbial biomass determined by the two methods correlated significantly (R^2^ = 0.55, P < 0.001). Combined with measurements of microbial biomass N (MBN), the latter was used for calculating microbial C-to-N ratio, whereas the former was used to calculate microbial specific respiration as both basal respiration and substrate-induced respiration were measured from the same soil sample (see below).

For measurement of microbial biomass by chloroform fumigation extraction (MBC_CFE_) two subsamples of 7 g were taken from each soil sample, one was fumigated with chloroform vapour for 24 h, while the other remained unfumigated. Fumigated and unfumigated samples were extracted with 40 ml 0.5 M K_2_SO_4_ with agitation for 30 min, the extracts were filtered and frozen. Total C and N in the extracts was measured by dry combustion in a DIMA-TOC 100 Analyzer (Dimatec, Essen, Germany). MBC_CFE_ was calculated as [(total C in fumigated soil)—(total C in non-fumigated soil)] / 0.45 [40]. MBN was calculated as [(total N in fumigated soil)–(total N in non-fumigated soil)]/0.54 [41]. Gravimetric soil water content was measured by drying subsamples at 105°C for 48 h. Microbial biomass C-to-N ratio was determined from data on soil microbial biomass C and N [41–43].

Microbial basal respiration was measured using an O_2_ microcompensation apparatus [44]. O_2_ consumption of soil microorganisms in fresh soil equivalent to 3.5 g dry weight was measured at 22°C over a period of 24 h. Basal respiration [μL O_2_ g^-1^ dry soil h^-1^] was calculated as mean of the O_2_ consumption rates of hours 14 to 24 after the start of measurements. Substrate-induced respiration [45] was determined by adding D-glucose to saturate catabolic enzymes of microorganisms according to preliminary studies (4 mg g^-1^ dry soil solved in 400 μL deionized water). Maximum initial respiratory response (MIRR; [μL O_2_ g^-1^ dry soil h^-1^]) was calculated as mean of the lowest three O_2_ consumption values within the first 10 h after glucose addition. MBC_SIR_ [μg C g^-1^ dry soil] was calculated as 38 × MIRR [46]. Data on microbial basal respiration and MBC_SIR_ were used to calculate microbial specific respiration (metabolic oxygen quotient; [μL O_2_ mg^-1^MBC h^-1^]) as a measure of microbial C use efficiency by dividing basal respiration by MBC_SIR_ [47]. MBC_SIR_ was used to analyze the response of microbial biomass C to experimental treatments, as done in previous studies of the Jena Experiment [14].

### Statistical analyses

Data (except microbial C-to-N ratio) were log-transformed to meet the requirements of parametric statistical tests. Plant species richness was log-transformed to linearize the saturating relationship between plant diversity and soil microbial properties [19]. Effects of block, plot, (log-transformed) plant species richness, plant functional group richness, fertilization, and presence of grasses, tall herbs, small herbs, and legumes as well as interactions between plant community factors and fertilization were analyzed by sequential split-plot general linear models (GLM, type I sum of squares). Block was fitted first in our model. As we focused on plant diversity effects, plant species richness or functional group richness were always fitted before presence/ absence of single plant functional groups. These were followed by plot, fertilization and the respective interactions between fertilization and plant community factors. F-values given in the results refer to those where the respective factor within each category (e.g., plant diversity or presence of plant functional groups) was fitted first [48]. The effects of block and plant community factors were tested against plot in order to avoid pseudo-replication, whereas fertilization and interactions were tested against the total error. Multiple comparisons of means were conducted using Tukey’s honest significant difference test. We did not correct for multiple statistical tests considering the mathematical and logical argumentation by Moran [49]. Statistical analyses were performed using SAS 9.3 (SAS Institute, Cary, USA). Regressions between microbial C-to-N ratio as well as aboveground plant biomass and microbial properties were carried out using Sigmaplot 10.0 (Systat Software Inc., San Jose, USA).

## Results

**Fig 1:**
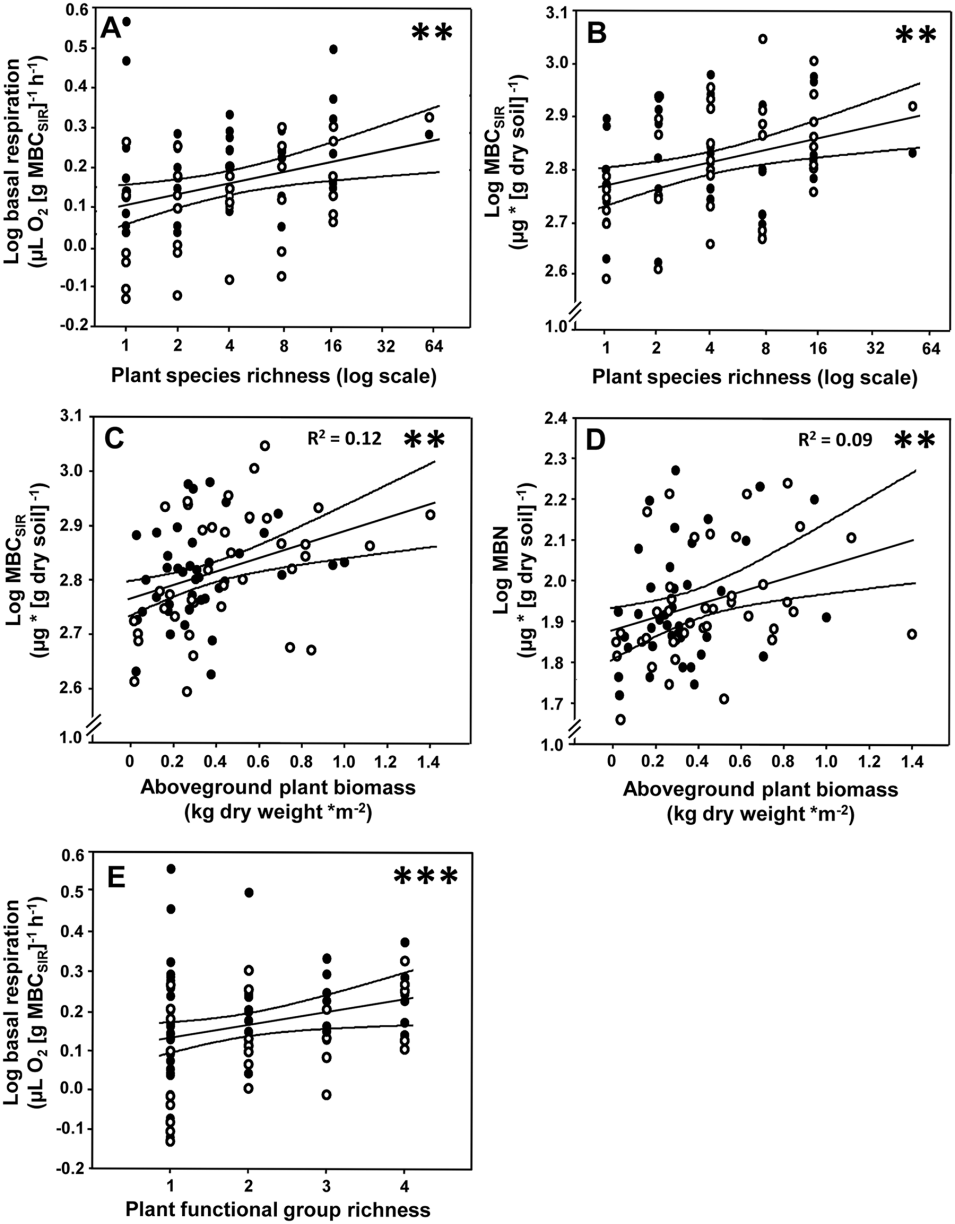
Effects of plant diversity and shoot biomass on microbial basal respiration and soil microbial biomass C and N. Effects of plant species richness on (A) basal respiration and (B) soil microbial biomass C. (C) Relationships between aboveground plant biomass (dry weight) and soil microbial biomass C, and (D) between aboveground plant biomass and soil microbial biomass N. (E) Effects of plant functional group richness on basal respiration. Note log scale of y-axes. Black dots are measures from unfertilized plots, white dots from fertilized plots. Regression lines with 95% confidence bands. Asterisks indicate significant differences (**p≤0.01, ***p≤0.001).

Soil water content and aboveground plant biomass were significantly increased by plant species richness (F = 13.12, P<0.001, R^2^ = 0.16, and F = 58.45, P< 0.001, R^2^ = 0.43, respectively). Moreover, plant species richness significantly increased basal respiration and MBC_SIR_ (Fig 1a and 1b; Table 1). Aboveground plant biomass and soil water content correlated positively with MBN (plant biomass: F = 8.31, P<0.01, R^2^ = 0.09,Fig 1c; soil water: F = 89.35, P<0.001, R^2^ = 0.53) and MBC_SIR_ (plant biomass: F = 10.88, P<0.01, R^2^ = 0.12, Fig 1d; soil water: F = 47.18, P<0.001, R^2^ = 0.37). Nevertheless, plant diversity effects on soil microbial respiration and MBC_SIR_ remained (marginally) significant even if accounting for the effect of aboveground plant biomass (respiration: F = 17.77, P<0.001; microbial biomass: F = 7.40, P<0.01) or soil water content (respiration: F = 5.53, P<0.05; microbial biomass: F = 3.23, P<0.1), indicating that plant diversity effects on soil microbial properties cannot be fully explained by aboveground plant biomass production and soil water content. Plant functional group richness significantly enhanced basal respiration, but only when fitted before plant species richness (Fig 1e).

**Table 1:** Fertilization and plant community effects on soil microbial properties.

	logBR			logMBC_SIR_			logqO_2_			logMBN			C-to-N ratio		
	**d.f.**	**F**	**P**	**d.f.**	**F**	**P**	**d.f.**	**F**	**P**	**d.f.**	**F**	**P**	**d.f.**	**F**	**P**
**Block**	1, 33	4.8	**0.0366**	1, 33	16.1	**0.0003**	1, 33	3.7	0.0621	1, 32	18	**0.0002**	1, 32	7.9	**0.0083**
**logSR**	1, 32	12	**0.0015** ↑	1, 32	8.21	**0.0073** ↑	1, 32	1.1	0.2988	1, 32	2.5	0.122	1, 32	0	0.8984
**FR**	1, 32	6.9	**0.0132** ↑	1, 32	3.04	0.0906	1, 32	1.7	0.2088	1, 32	0.6	0.458	1, 32	0.2	0.689
**GR**	1, 32	0.5	0.4994	1, 32	0.44	0.5133	1, 32	2.7	0.1116	1, 32	3.1	0.087	1, 32	**5**	**0.0327** ↑
**LEG**	1, 32	7.4	**0.0103** ↓	1, 32	1.56	0.2203	1, 32	4	**0.0552** ↓	1, 32	0.3	0.601	1, 32	**6.5**	**0.0156** ↓
**TH**	1, 32	0.3	0.5856	1, 32	<0.01	0.9706	1, 32	0.4	0.5182	1, 32	1.5	0.238	1, 32	0.2	0.6669
**SH**	1, 32	9	**0.0051** ↑	1, 32	7.43	**0.0103** ↑	1, 32	0.4	0.5416	1, 32	1.9	0.179	1, 32	0.2	0.643
**Plot**	32, 33	1	0.4781	32, 33	2.36	**0.0083**	32, 33	1	0.4507	32, 32	5.8	**<.0001**	32, 32	6.1	**<.0001**
**FERT**	1, 32	25	**<.0001** ↓	1, 32	0.22	0.6418	1, 32	45	**<.0001** ↓	1, 32	0	0.943	1, 32	0.4	0.5202
SR * FERT	1, 32	2.7	0.1073	1, 32	2.62	0.1152	1, 32	0.7	0.3947	1, 32	2.2	0.144	1, 32	0	0.9049
FR * FERT	1, 32	2.8	0.1068	1, 32	0.53	0.4738	1, 32	2.4	0.1283	1, 32	1.5	0.237	1, 32	0.1	0.8107
GR * FERT	1, 32	2.1	0.1545	1, 32	<0.01	0.9568	1, 32	3.3	0.0773	1, 32	0	0.892	1, 32	2.1	0.1619
LEG * FERT	1, 32	5	**0.0322**	1, 32	0.01	0.9343	1, 32	**7.8**	**0.0085**	1, 32	0.8	0.394	1, 32	0.1	0.7176
TH * FERT	1, 32	0.1	0.7453	1, 32	0.07	0.7912	1, 32	0	0.8335	1, 32	0.2	0.656	1, 32	0.3	0.5655
SH * FERT	1, 32	0.3	0.5753	1, 32	0.1	0.7579	1, 32	0.2	0.6323	1, 32	0.1	0.727	1, 32	0.4	0.5201
**d.f. error**	33			33			33			32			32		
**d.f. model**	46			46			46			46			46		
**F-statistic**	2.24			3.49			2.22			7.48			6.43		

GLM (type I sum of squares) table of F-values for effects of block, plot, fertilization (FERT), log-transformed plant species richness(logSR), plant functional group richness (FR), presence of grasses (GR), legumes (LEG), small herbs (SH) or tall herbs (TH) and the respective interactions between fertilization and plant community properties on log-transformed data of microbial basal respiration (logBR), microbial biomass C (logMBC_SIR_), microbial specific respiration (logqO_2_), and microbial biomass N (log MBN) and un-transformed microbial C-to-N ratio. F-values refer to those where the respective factor was fitted first within its category (plant diversity or presence of plant functional groups). d.f. = degrees of freedom; F = F-value; P = p-value.

^↑ / ↓^ = increase/decrease with increasing diversity level or in presence of the respective plant functional group or treatment. Significant effects (P≤0.05) are given in bold.

**Fig 2:**
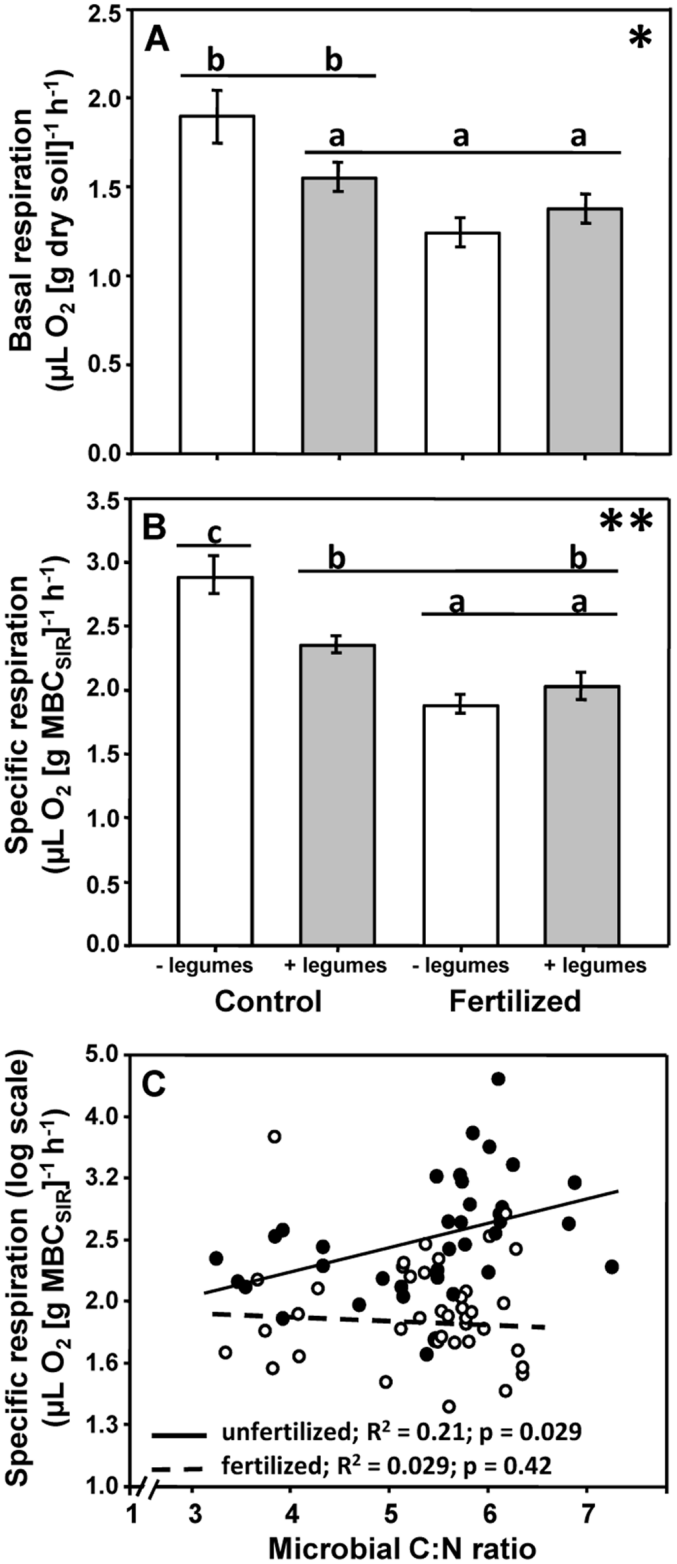
Interactive effects of the presence of legumes and fertilization on microbial basal respiration and specific respiration, and correlation between microbial C-to-N ratio and microbial specific respiration in fertilized and unfertilized plots. (A) Interactive effects of the presence of legumes and fertilization on soil microbial basal respiration and (B) specific respiration. Means with standard error bars. Different letters indicate significant differences (*p≤0.05; **p≤0.01). (C) Regressions between soil microbial C-to-N (C:N) ratio and specific respiration in unfertilized plots (black line, black dots) and fertilized plots (dashed line, white dots) with coefficients of determination and p-values of slopes.

The interaction between the presence of legumes and fertilization significantly affected basal respiration and specific respiration: legumes reduced basal and specific respiration in non-fertilized plots, but increased both of these variables in fertilized plots (Fig 2a and 2b). As indicated by regression analyses specific respiration increased significantly with increasing microbial C-to-N ratio in non-fertilized but not in fertilized plots (Fig 2c).

**Fig 3:**
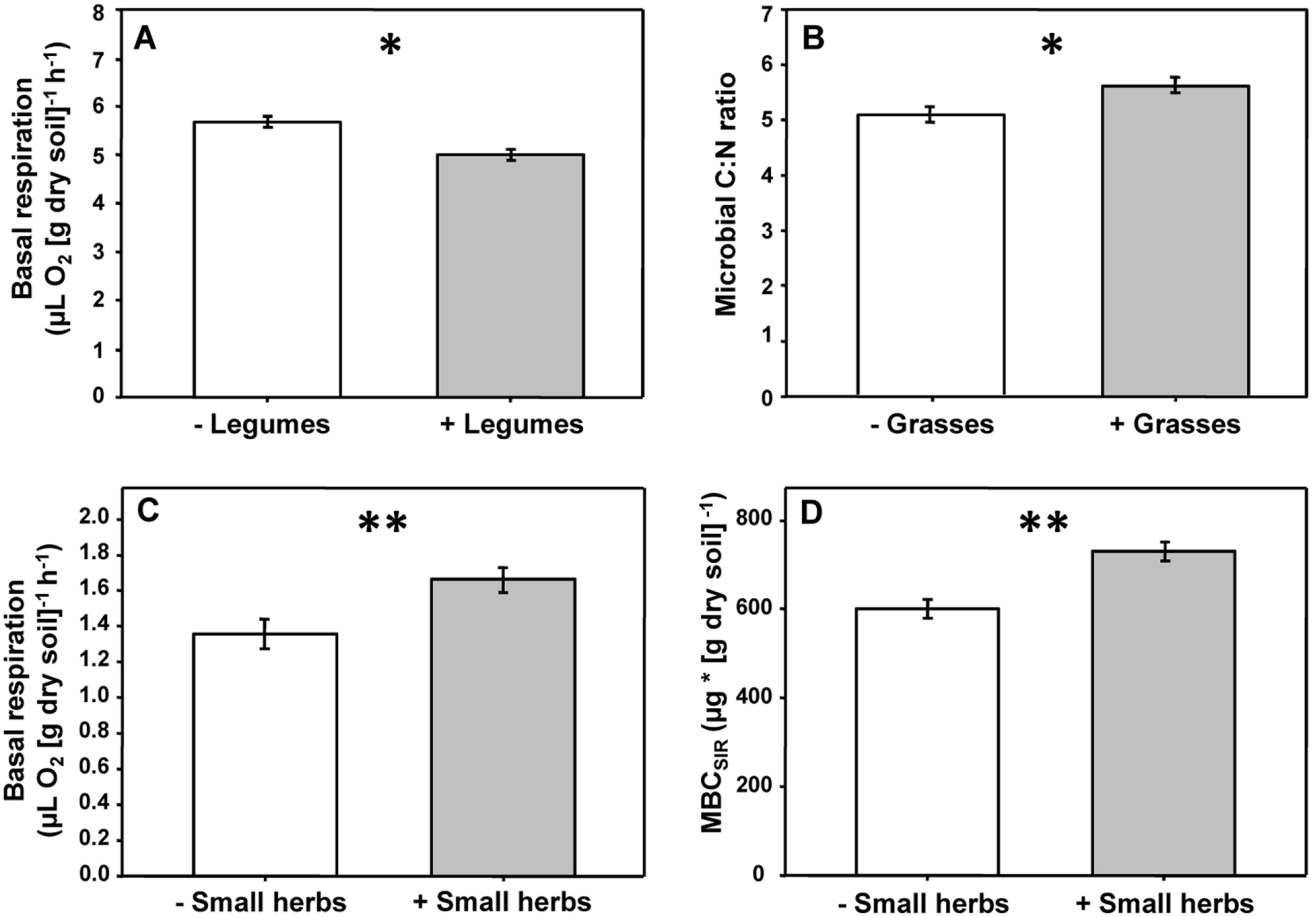
Effects of plant functional groups on microbial properties. Effects of (A) legumes and (B) grasses on microbial C-to-N (C:N) ratio. Effects of small herbs on (C) basal respiration and (D) soil microbial biomass C. Asterisks indicate significant differences (*p≤0.05, **p≤0.01). Means with standard error bars.

The presence of legumes significantly decreased microbial C-to-N ratio (-12%; Fig 3a), while the presence of grasses significantly enhanced it (+9%; Fig 3b). Further, the presence of small herbs significantly increased basal respiration (+18%; Fig 3c) and soil MBC_SIR_ (+18%; Fig 3d). Note that effects of presence of different plant functional groups are effects with plant diversity factors (plant species richness and plant functional group richness) fitted before plant functional groups. Neither MBN nor microbial C-to-N ratio were significantly affected by plant diversity measures or by fertilization.

## Discussion

We investigated effects of plant community properties and fertilization on soil microbial biomass, respiration and C use efficiency. Plant community properties significantly affected soil microbial activity (respiration) and biomass, while fertilization affected microbial activity and C use efficiency (specific respiration). In contrast to our hypothesis, effects of plant diversity (species and functional group richness) were independent of fertilization. Our results highlight the importance of plant diversity for increased basal respiration and soil microbial biomass and are in line with previous studies [4,12,22]. Plant functional groups, legumes, grasses, and small herbs contrastingly affected soil microbial properties. The significant correlation between soil water content and plant species richness as well as the weaker plant species richness effect when fitted after soil water content suggests that effects of plant diversity on soil microbial respiration and biomass are mediated, at least in part, by changes in soil water content [50,51]. Our microbial stoichiometry results (C-to-N ratio) suggest that legumes reduced N limitation of soil microorganisms, and that under N limitation microbial stoichiometry determines the functioning of soil microbial communities (as indicated by changes in microbial specific respiration). Additionally, the ratio between fungal and bacterial biomass may have shifted towards bacteria in the presence of legumes as fungal biomass is known to decrease in presence of legumes and with increased N input [52].

In accordance with our hypothesis 1, plant species richness significantly increased soil microbial biomass and basal respiration. Plant diversity effects on soil microbial properties remained significant even after accounting for the effect of aboveground plant biomass. This suggests that plant diversity impacted soil microbial functioning via mechanisms not directly related to aboveground plant productivity. Plant functional group richness increased microbial basal respiration, but did not significantly affect the other soil microbial parameters measured. The lack of effects of plant diversity on microbial specific respiration was unexpected, as Wardle and Ghani [53] showed microbial specific respiration to decrease in more stable ecosystems such as those with high plant diversity [54]. High diversity plant communities are likely to provide high amounts of resources to decomposer communities [9]. Aboveground plant biomass correlated positively with MBC_SIR_ and MBN despite plants were cut and residues were removed from the plots after mowing, suggesting that enhanced C and N availability to soil microorganisms in high diversity communities was due to increased amounts of root-derived resources [55]. Microbial communities in the rhizosphere are known to heavily rely on root exudates [56] and other rhizodeposits [57]. Further, more constant C and N inputs into the soil and high plant coverage in high diverse plant communities [58,59] as well as more constant and favorable soil moisture [27] may have contributed to higher soil microbial respiration and biomass at high plant diversity [14].

Hypothesis 2 suggested plant functional groups to differently affect soil microbial properties, due to group-specific plant functional traits [14,38,60]. Supporting this hypothesis, the presence of legumes significantly reduced basal respiration, specific respiration, and microbial C-to-N ratio, while small herbs significantly enhanced soil microbial basal respiration and biomass. The fact that legumes did not alter soil microbial biomass is in line with findings of Zak et al. [12], but contrasts with other plant diversity studies in experimental grasslands (e.g., [21,61]). Eisenhauer et al. [14] found increased soil microbial biomass in the presence of legumes at the same field site four years before the present study, but this effect disappeared two years later, indicating a change in the effects of certain plant functional groups on microbial properties over time. N_2_ fixation by legumes requires high amounts of phosphorus [62], potentially leading to stronger P limitation of soil microorganisms as compared to plant communities without legumes. Indeed, Oelmann et al. [63] found legumes to reduce labile inorganic P compounds at our study site. Thus, competition for P between soil microorganisms and legumes may explain the missing legume effect on soil microbial biomass in this study [64]. Lower microbial C-to-N ratio in the presence of legumes indicates improved nitrogen supply of soil microorganisms due to N_2_ fixation by legumes. Therefore, the reduced microbial specific respiration in the presence of legumes likely was due to improved C use efficiency induced by increased N supply.

In contrast to legumes, the presence of grasses increased microbial C-to-N ratio and specific respiration, indicating reduced microbial C use efficiency. Grasses are characterized by higher tissue C-to-N ratios than other plant functional groups and by building dense fibrous root systems with high specific root length [65,66]. Thus, grasses likely enhance microbial activity and biomass by providing large amounts of rhizodeposits [67]. However, we suggest grasses to force soil microorganisms to invest more energy into metabolic activity to alleviate N limitation, resulting in competition for N between soil microorganisms and plants [34]. Reduced soil water content in the presence of grasses likely aggravated the competition between plants and microorganisms for capturing N [34,68]. Small herbs are generally shallow-rooting, with most roots in the soil layer sampled in the present study. Presumably, increased soil microbial activity and biomass in the presence of small herbs in our study were due to increased rhizodeposition in the topsoil [69–71].

In contrast to hypothesis 3, fertilization did not affect soil microbial biomass. In earlier studies effects of fertilization on soil microbial biomass have been found to be positive [72,73], negative [33,74] or neutral [75], suggesting that higher plant productivity due to fertilization does not uniformly translate into increased soil microbial biomass. A number of mechanisms may explain the lack of fertilizer effects on soil microbial biomass. First, the removal of the aboveground biomass after mowing prevented aboveground litter from entering the soil. Second, fertilization generally reduces plant resource allocation to belowground structures, resulting in reduced root biomass [76–78], and hence, reduced root deposits serving as resources for microorganisms. Third, as the response of soil microbial communities to changes in plant community composition has been shown to lag behind by several years [14], our two-year study may have been too short to uncover the full effects of fertilization on soil microorganisms [33]. Soil microbial activity may have responded earlier to fertilization than microbial biomass, due to fertilizer-mediated changes in rhizodeposition [4] or reduced root exudation [32].

Fertilization superimposed the negative legume effect on basal and specific respiration. Legumes are known to negatively respond to N fertilization as they may be outcompeted by grasses starting to grow earlier in the season and having a more efficient root system for nutrient uptake [79,80]. Notably, both legumes and fertilizer addition decreased microbial respiration, but the underlying mechanisms are likely to be different: legumes decreased microbial activity probably by improving organic N supply, while fertilization presumably acted through decreasing rhizosphere priming effects [81] and simultaneously through provision of inorganic N used by microorganisms [82]. Indeed, earlier studies in forest soils also found N amendment to decrease soil microbial activity [76,83].

Although it remains elusive whether decreased specific respiration was induced by inhibition of microbial metabolism [33] or by increased microbial C use efficiency [53], we assume the latter to be more likely as fertilization alleviates N limitation of microorganisms with high C-to-N ratio. In non-fertilized plots, microbial C-to-N ratio was positively correlated with specific respiration, while this was not the case in fertilized plots, indicating that soil microorganisms at the field site of the Jena Experiment are N limited as indicated in earlier studies [14]. Hence, our results demonstrate microbial stoichiometry to be a powerful indicator of soil microbial functioning in N limited systems [84]. In addition, microbial C-to-N ratios at our study site are close to that of bacteria (i.e., 5:1 [85]), suggesting that bacteria rather than fungi were responsible for the observed responses [50].

Contrary to hypothesis 4, fertilization did not strengthen the effects of plant diversity (species richness and functional group richness) on soil microbial respiration or biomass. Except for the interaction between legumes and fertilizer addition discussed above, fertilization and plant diversity did not in an interactive way affect any soil microbial parameters measured. This contrasts earlier studies reporting the addition of N to increase effects of plant diversity on ecosystem functioning [25,86], or to induce positive biodiversity-ecosystem functioning relationships [87]. At the Jena Experiment field site Weigelt et al. [37] found N fertilization to slightly increase effects of plant diversity on primary production. Overall, this suggests that plant diversity and fertilization act through decoupled mechanisms on microbial properties with the effects being independent of N fertilizer-induced increase in plant productivity in more diverse plant communities. Alternatively, the weak interactive effect of plant diversity and fertilization on plant biomass production may not be strong enough to cascade to changes in soil microbial respiration and biomass.

## Conclusions

Overall, plant diversity beneficially affected soil microorganisms, likely due to changes in rhizodeposition, plant productivity, and soil moisture. Our results underline the importance of plant functional groups, in particular legumes, for soil microbial functioning and stoichiometry. Thus, promoting high plant diversity in managed grasslands, by including certain plant functional groups, is likely to beneficially affect microbially-driven ecosystem functions such as decomposition and element cycling. Generally, effects of plant diversity and fertilization were independent, while the effect of legumes on microbial C use efficiency was modified by fertilization. Both legumes and fertilization alleviated N limitation of soil microorganisms, but this likely was due to different mechanisms with legumes acting via provisioning of organic N, and fertilization acting via provisioning of inorganic N and decreasing rhizosphere priming effects. Our results suggest that both fertilizer application and the presence of legumes reduce soil microbial N limitation, and thereby modulate soil microbial stoichiometry and functioning. To mechanistically understand the observed response of microorganisms root-derived resources need closer investigation.

## Supporting Information

S1 Fig Realized species richness in fertilized and unfertilized subplots. Number of realized species in unfertilized vs. fertilized subplots for (A) all species, (B) grasses, (C) small herbs, (D) tall herbs and (E) legumes. Given are means (+/- standard error) recorded in 2008 on plots with sown diversity levels of 1, 2, 4, 8 and 16 plant species. Species richness was derived from species specific frequency measurements in 30 quadrats of 10 x 10 cm in size within the core area of 1 m^2^ of treated subplots.
(TIF)

S1 Dataset Data on soil microbial parameters, soil moisture, aboveground plant biomass and predictor variables used for the statistical analyses. (XLS)

S2 Dataset Data on sown and realized plant species numbers on the experimental plots of the Jena Experiment in 2008. (XLS)
