# Supplementary figures and images for: Effects of Plant Diversity, Functional Group Composition, and Fertilization on Soil Microbial Properties in Experimental Grassland
Source: PLoS One. 2015 May 4;10(5):e0125678. doi: 10.1371/journal.pone.0125678 (PMC4418810; doi:10.1371/journal.pone.0125678)

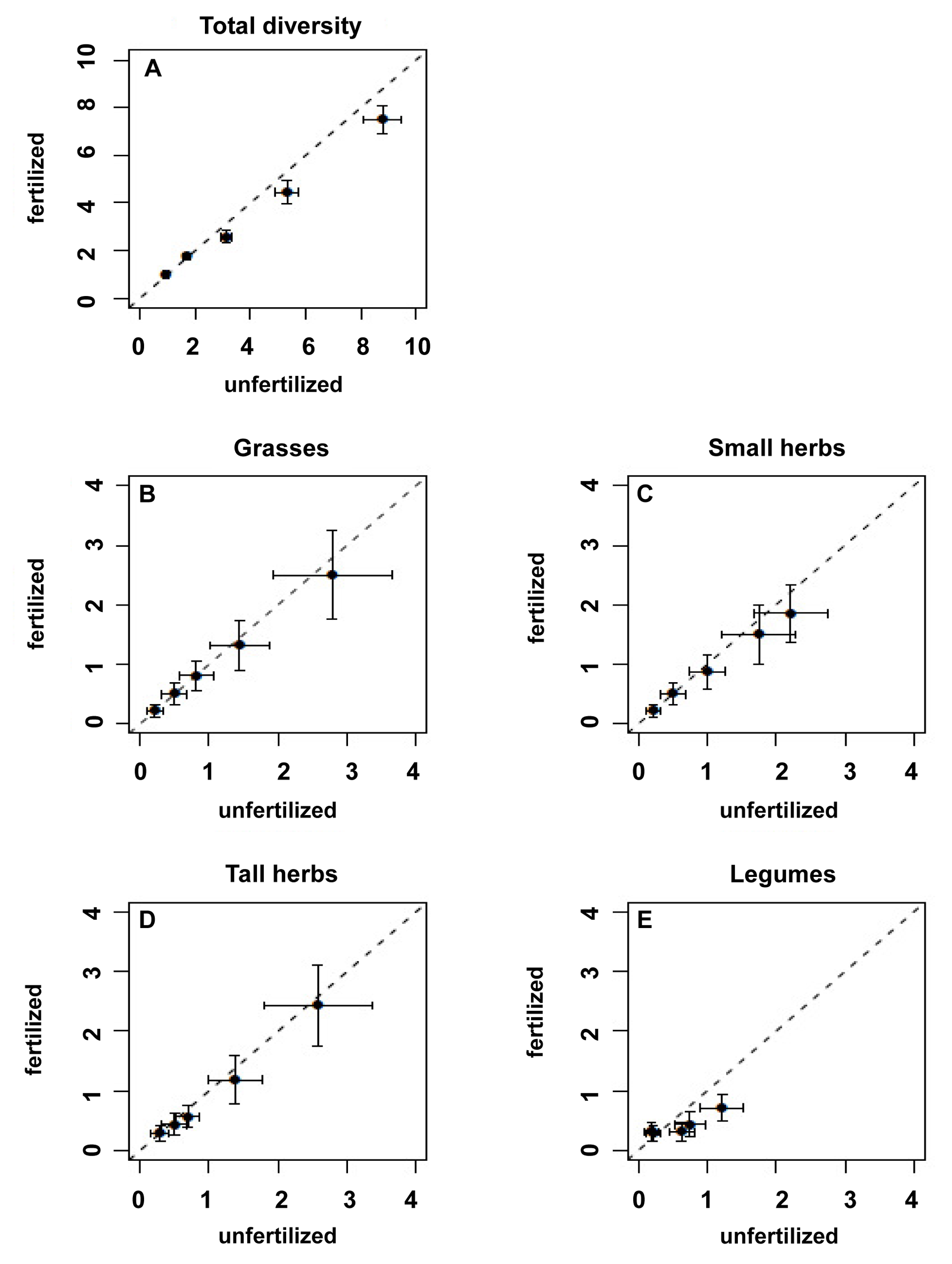

Supplement: S1 Fig — Number of realized species in unfertilized vs. fertilized subplots for (A) all species, (B) grasses, (C) small herbs, (D) tall herbs and (E) legumes. Given are means (+/- standard error) recorded in 2008 on plots with sown diversity levels of 1, 2, 4, 8 and 16 plant species. Species richness was derived from species specific frequency measurements in 30 quadrats of 10 x 10 cm in size within the core area of 1 m2 of treated subplots. (TIF) [file pone.0125678.s001.tif]
